# 'How to know what you need to do': a cross-country comparison of maternal health guidelines in Burkina Faso, Ghana and Tanzania

**DOI:** 10.1186/1748-5908-7-31

**Published:** 2012-04-13

**Authors:** Ulrika Baker, Göran Tomson, Mathias Somé, Bocar Kouyaté, John Williams, Rose Mpembeni, Siriel Massawe, Antje Blank, Lars L Gustafsson, Jaran Eriksen

**Affiliations:** 1 Department of Public Health Sciences, Division of Global Health (IHCAR), Karolinska Institutet, Nobels väg 9, SE-17177 Stockholm, Sweden; 2 Department of Learning, Informatics, Management and Ethics (LIME), Medical Management Centre (MMC), Karolinska Institutet, Berzelius väg 3, SE-17177 Stockholm, Sweden; 3 Centre de Recherche en Santé de Nouna (CRSN), BP 02, Nouna, Burkina Faso; 4 Navrongo Health Research Centre, P.O. Box 114, Navrongo, Ghana; 5 Department of Epidemiology and Biostatistics, Muhimbili University of Health and Allied Sciences (MUHAS), School of Public Health and Social Sciences, P.O. Box 65001, Dar Es Salaam, Tanzania; 6 Department of Obstetrics and Gynaecology, Muhimbili University of Health and Allied Sciences, School of Medicine, P.O. Box 65001, Dar-Es-Salaam, Tanzania; 7 Department of Clinical Pharmacology and Pharmacoepidemiology, Medizinische Klinik (Krehl Klinik), University Hospital of Heidelberg, Im Neuenheimer Feld 410, 69120 Heidelberg, Germany; 8 Department of Laboratory Medicine (LABMED)Division of Clinical PharmacologyKarolinska Institutet, Karolinska University Hospital, F58, 141 86, Stockholm, Sweden; 9 London School of Hygiene and Tropical Medicine (LSHTM), Keppel Street, London WC1E 7HT, UK

**Keywords:** CPGs, Health service delivery, Implementation, Information and communication technology (ICT), Maternal health, Quality improvement, Sub Saharan Africa, WHO

## Abstract

**Background:**

Initiatives to raise the quality of care provided to mothers need to be given priority in Sub Saharan Africa (SSA). The promotion of clinical practice guidelines (CPGs) is a common strategy, but their implementation is often challenging, limiting their potential impact. Through a cross-country perspective, this study explored CPGs for maternal health in Burkina Faso, Ghana, and Tanzania. The objectives were to compare factors related to CPG use including their content compared with World Health Organization (WHO) guidelines, their format, and their development processes. Perceptions of their availability and use in practice were also explored. The overall purpose was to further the understanding of how to increase CPGs' potential to improve quality of care for mothers in SSA.

**Methods:**

The study was a multiple case study design consisting of cross-country comparisons using document review and key informant interviews. A conceptual framework to aid analysis and discussion of results was developed, including selected domains related to guidelines' implementability and use by health workers in practice in terms of usability, applicability, and adaptability.

**Results:**

The study revealed few significant differences in content between the national guidelines for maternal health and WHO recommendations. There were, however, marked variations in the format of CPGs between the three countries. Apart from the Ghanaian and one of the Tanzanian CPGs, the levels of both usability and applicability were assessed as low or medium. In all three countries, the use of CPGs by health workers in practice was perceived to be limited.

**Conclusion:**

Our cross-country study suggests that it is not poor quality of content or lack of evidence base that constitute the major barrier for CPGs to positively impact on quality improvement in maternal care in SSA. It rather emphasises the need to prioritise the format of guidelines to increase their usability and applicability and to consider these attributes together with implementation strategies as integral to their development processes.

## Background

Effective interventions to reduce maternal mortality exist, but their implementation into programs rendering a substantial impact in the worst affected countries remains a challenge [1,2]. In 2008, an estimated 358,000 women died worldwide due to pregnancy-related causes of which 204,000 deaths occurred in in Sub Saharan Africa (SSA) [3]. In all three study countries presented in this paper--Burkina Faso, Ghana, and Tanzania--the magnitude of maternal mortality remains unacceptably high. Differences in maternal mortality ratio (MMR) estimates are however large, not only between but also within countries. This illustrates the well-recognised challenge of measuring and estimating maternal deaths in low-income settings with poor vital registration systems (Table 1) [3-5]. The highest estimated MMR for Tanzania at 790 per 100,000 live births translates to a lifetime risk of one in every 23 women [3]. In Burkina Faso, the corresponding MMR is estimated at 560 with a lifetime risk of dying from maternal causes of one in 28 women [3]. Ghana is currently the only country making progress towards millennium development goal 5 (MDG 5), which aims for a 75% reduction of the MMR between 1990 and 2015 [3]. It is also the country where statistics on maternal deaths vary least between data sources with MMR estimates of 350 and 409 [3,5].

**Table 1:** Maternal Mortality Ratio (MMR) estimates in the three countries using two different sources

Sources	MMR: number of maternal deaths/100,000 live births (uncertainty range)		
	
	Burkina Faso	Ghana	Tanzania
Trends in maternal mortality: 1990-2008. Estimates developed by WHO, UNICEF, UNFPA and The World Bank. 2010[3].	560 (330 to 950)	350 (210 to 600)	790 (470 to 1300)

Maternal mortality for 181 countries, 1980 to 2008: a systematic analysis of progress towards Millennium Development Goal 5. Lancet, 2010[5].	332 (208 to 522)	409 (248 to 633)	449 (273 to 721)

It is widely accepted that the improvement of maternal health relies on strengthening the entire health system [6,7]. Encouraging women to deliver in health facilities, and thus increasing the proportion of deliveries with skilled attendance, has been proposed as a main priority and a key indicator by which to measure progress towards MDG 5 [1,8]. However, less focus has been given to the quality of care given by skilled attendants and to the resources with which they have to work [9]. In a four-country comparison of health system factors influencing maternal health services [6], it is suggested that 'the context in which staff work, the quality of human resources management, and issues around healthcare worker motivation are as important as whether staff are present or not' [6].

Medical care provided to patients in low-income countries is often of poor quality and the deficiencies in health worker performance a threat to service delivery in many settings [9-11]. Low quality of care may also be a major deterrent of pregnant mothers resulting in underuse of services [4]. While the overwhelming lack of human resources for health is often considered a main reason, a recent study from rural Tanzania did not find any association between work load and level of effort of health workers, suggesting that an increase of the work force alone is unlikely to improve quality of care [12]. Instead, increasing importance and focus is given to the role of poor motivation and the significant 'know-do gap' in health worker performance [6,10,13,14].

One strategy commonly applied to increase the skill levels of health workers and improve the quality of health service provision is the use of clinical practice guidelines (CPGs), which have become increasingly common worldwide since the early 1990s [15-18]. Considerable resources are dedicated to their development and production while implementation, uptake, and use by health workers remains challenging, thus limiting their potential to improve quality of care [11,18]. A review from 2008 showed small beneficial effects of passively disseminated guidelines (printed educational materials) on professional practice [19], and it is today widely acknowledged that the success of guideline implementation relies on multi-facetted interventions including training, follow-up, and supervision as a minimum package [11,17,20]. An intervention study from 2007 illustrated this by showing sustained improvements in quality of care and adherence to family planning guidelines by health workers in Kenya after introducing intensive 'cascade training' and supportive supervision [17]. Recent research has focused on factors associated with successful guideline implementation including attributes such as their evidence base, format, clarity of advice, complexity, and level of involvement of stakeholders [16,18,21]. The value of using information and communication technology (ICT) to improve guideline implementation has become well-established in high-income countries and interventions to trial its use in low-income settings increasingly common [20,22-26]. A recent study from Kenya, for example, showed promising results on adherence to antiretroviral treatment for HIV through the use of mobile phone text messages [26].

Cross-country analyses of health policies and programs can yield insights and contribute to the development of new theories [27]. Comparisons across different contexts have the potential to reveal common themes and domains that cannot be explored by single country case studies. At the same time, such comparisons are both time consuming and resource intense, and examples from the literature are sparse [27,28]. This study is part of QUALMAT (Quality of Maternal and Prenatal Care: Bridging the Know-do Gap), an EU-funded collaborative research project between the Centre de Recherche en Santé de Nouna (Burkina Faso), Ghent University (Belgium), Heidelberg University (Germany), Karolinska Institutet (Sweden), Muhimbili University of Health and Allied Sciences (Tanzania), and Navrongo Health Research Centre (Ghana) [29]. One of the objectives of the QUALMAT project is to develop and implement a computerised decision support system (CDSS) for health workers to use in maternal care in rural Burkina Faso, Ghana, and Tanzania. Its purpose is to work both as an incentive to better motivate health workers and to improve quality of care through increased adherence to guideline recommendations. We conducted cross-country comparisons of the national CPGs for maternal healthcare in Burkina Faso, Ghana and Tanzania. The objective was to compare factors related to CPG use including their content compared with World Health Organization (WHO) guidelines, their format, and their development processes. The data collected also yielded an opportunity to assess key stakeholders' perceptions of CPGs' availability and use in practice. The study presented in this paper provided background information for the development and adaptation of the CDSS to the national contexts. The findings were used in a workshop in which the content of country specific versions of the tool was developed. The focus was therefore on format and content of the CPGs. In addition to providing background information for the CDSS, the overall purpose was to further the understanding of how to increase the potential of CPGs to improve quality of care for mothers in SSA. With its focus on a three-country comparison, this study fills an important gap.

## Methods

### Study design

The study is a multiple case study design consisting of cross-country comparisons of three case studies [6,30,31] using: document review to compare the content of national CPGs with WHO recommendations and to assess the format of guidelines; and key informant interviews to explore knowledge of national maternal health CPGs, their development processes, relation to WHO recommendations, and perceptions of their availability and use by health workers in practice.

### Study setting

The study was carried out in two West African countries, Burkina Faso and Ghana, and one East African country, Tanzania. In all countries, an intervention and a non-intervention district have been chosen where the QUALMAT project will be conducted in 2009 to 2014 in rural primary healthcare centres providing maternal and neonatal care. All three study countries suffer from a critical shortage of health workers [32,33], but variations are large with the density of nurses and midwives in Ghana five times that of Tanzania [33]. Skilled attendance at delivery is similar with national averages around 50% in all three countries [33].

### Data collection

Data collection was carried out between September 2009 and March 2010. National CPGs for maternal care with contents corresponding to the WHO guideline 'Pregnancy Childbirth Postpartum and Newborn Care: A Guide for Essential Practice' (PCPNC) sections B9-E, the sections relevant to the CDSS development, were identified and collated (see further explanation under document review). This was done with the help of local research partners and co-authors (MS, BK, JW, RM, SM), as well as key informants at the ministries of health and other organisations involved in the development of national guidelines for maternal health. Several rounds of crosschecking were done to make sure that all the relevant national maternal health CPGs had been identified.

Semi-structured interviews were held with a total of 38 key informants in the three countries. The interview guide had three sections which covered information on: 1) national CPGs for maternal health; 2) motivation and incentive policies for human resources; and 3) current maternal health projects and initiatives in the study areas (see Additional file 1 for interview guide for topics one and three). Ten of the key informants worked in human resources and administration and were not asked questions regarding topics one and three. Only the 28 interviews covering information on CPGs for maternal health (Table 2) were therefore included in the study presented in this paper. The sampling was mainly purposive where an initial list of key informants [34] representing relevant government departments, international and national non-governmental organisations (NGOs) and with knowledge of maternal health guidelines, was prepared by the research teams in each country headed by RM, JW and MS. Each key-informant was also asked about other key stakeholders, but the majority of the ones mentioned were already on the initial lists. Because the objective was to identify national guidelines and policies, the majority of interviews took place at the central level, *e.g*., government departments and ministries as well as at national offices for NGOs and United Nations (UN) organisations. In Burkina Faso and Ghana, however, the research teams were based near the intervention and control districts, and therefore took the opportunity to interview a few key informants also at the regional and district level as well as at a few health facilities (Table 2) [35]. Interviews in Tanzania and Ghana were conducted in English and recorded and transcribed by the first author (UB) and two research assistant (MD and GH, see acknowledgements), respectively. In Burkina Faso, interviews were conducted by one of the co-authors (SM) in French and later translated into English.

**Table 2:** Key informant characteristics

Key informant category	Burkina Faso	Ghana	Tanzania	TOTAL
**Government level**	3	1	1	5

**Regional level**	1	1		2

**District level**	1	2		3

**Health Facility level**	5	2		7

**NGOs including WHO and other UN agencies, Faith based and Civil society organisations**	1	4	6	11

**TOTAL**	11	10	7	28

### Data analysis

#### Conceptual framework for cross-country comparison of clinical practice guidelines

**Figure 1:**
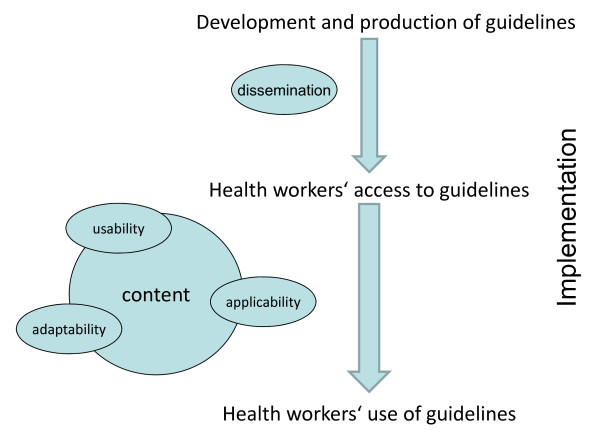
**Conceptual framework for cross-country comparison of clinical practice guidelines (CPGs)**. The framework describes the process from development and production to health workers' access to and use of CPGs. It emphasises three selected features (from Gagliardi *et al.*, 2011 [21]) of CPGs related to their implementability and use by health workers in practice: usability, applicability, and adaptability.

A conceptual framework describing the implementation process from development and production to health workers' access to and use of CPGs was constructed to aid analysis and discussion of the results in this paper (Figure 1). It emanated both from the document review and analysis of interviews, as well as from the adaptation of a recently published guidelines' implementability framework by Gagliardi *et al. *[21]. While Gagliardi and colleagues present a comprehensive model with eight overarching domains related to guidelines' implementability, our framework is purposefully limited with a focus on health workers as the users of guidelines. In this context, usability refers to the user-friendliness of a guideline and includes aspects such as ease of navigation and the format of evidence and recommendations such as tables and algorithms [21]. Applicability represents the degree to which the guideline can be used and applied by health workers for the management of individual patients [21]. Adaptability describes the availability of different versions of a guideline such as electronic formats or leaflets aimed at different users [21].

A recent paper [28] presents desirable features for a framework aimed at cross-country analysis. These include the need for such a framework to: offer insights into a broad variety of contexts; address the same problems in the same way whilst allowing for contextual differences; and use concepts sensitive to both quantitative and qualitative differences [28]. We believe that our framework considers all of these features. The cross-country comparison thus focuses on the description of contrasting and common features of the themes and categories emerging from the data analysis from each country, linking them to the elements of this framework. This is a methodology commonly applied for cross-country comparisons [6,30].

### Document review

The WHO PCPNC guideline [36] is part of the WHO tool kit for the 'Integrated Management of Pregnancy and Childbirth.' It provides generic norms and standards to be adapted for skilled health service providers at the primary care level. It is a guide supporting clinical decision making for health workers engaged in antenatal care, delivery, postpartum, post-abortion, and emergency care, as well as care for newborns immediately after delivery and during the first week of life. The introduction to the guide states that 'correct use of this guide should help reduce the high maternal and perinatal mortality rates [...], thereby making pregnancy and childbirth safer' [36]. This guideline is an internationally recognised tool on which national guidelines are commonly based, and was therefore chosen by the QUALMAT project as a gold standard on which to base the development of the CDSS and to be used for comparison with national guidelines.

All identified guidelines with contents corresponding to the WHO PCPNC sections B9-E were included in the data analysis [36]. Each guideline was systematically compared [37] with the WHO PCPNC, which is divided into chapters A to N with different numbered sub-sections. For this, we used a data extraction checklist (see Additional file 2) of key contents in the sections relevant to the CDSS development (sections B9-E). The data extraction from national guidelines was done according to sub-elements of these sections, which included: emergency care in pregnancy; bleeding in early pregnancy; antenatal care; labour; delivery; and postpartum care (see Additional file 2 for full contents of data extraction check-list). Deficiencies and discrepancies between national CPGs and the WHO PCPNC were noted down in a grid.

The format of guidelines was then examined to make an assessment of their usability and applicability, both attributes believed to influence their use in practice. It is also a primary objective of the CDSS in QUALMAT to ensure a high level of these attributes [21,38]. Usability and applicability of each guideline included were graded as high, medium, or low. Usability was assessed by: indexing and ease of navigation; format of text (narrative, check lists); and availability of treatment/management algorithms. A guideline was deemed as having a high degree of usability if it was easy to navigate and contained comprehensive checklists and algorithms. Guidelines with text in a narrative format, poor indexing, and no algorithms were deemed as having a low degree of usability, and guidelines falling between these categories as having a medium degree of usability. Applicability was assessed by: the availability of treatment/management algorithms to guide decision-making for individual patients; and availability of Partograph to monitor labour. Applicability was deemed high for guidelines containing clear treatment/management algorithms for different levels of care. Guidelines containing no algorithms but with a Partograph were deemed as having a medium degree of applicability, and guidelines without both algorithms and Partograph as having low applicability. The guideline review was primarily carried out by JE with additional input from UB and SM, and complemented by discussions with maternal health specialists in Burkina Faso and Ghana (see Acknowledgements).

### Analysis of interviews

The qualitative analysis of interviews was carried out with the help of Nvivo9^® ^software [39] and applied thematic content analysis [35,40]. Interview transcripts were initially sorted into content areas, and sections containing information on guidelines were further analysed to identify themes. These were partly framed by the initial interview guide and partly by the information emerging from analysis of the interview transcripts. Within each theme, further coding of information took place revealing several categories [35,40]. Data from each country were initially analysed independently. Themes and categories were then harmonised between the countries and in the last stage, a cross-country comparison of the results from interview data was performed. Analysis of interviews was carried out jointly by UB, JE, and GT.

### Ethical considerations

Verbal consent was obtained from all participating key informants who were informed that their participation was voluntary and that they could withdraw from the study at any time without stating a reason and without any implications. Ethical clearance was granted by: the Institutional Review Board at the Navrongo Health and Research Centre (Ethics Approval ID NHRCIRB 085) for Ghana; the Muhimbili University of Health and Allied Sciences Ethical Review Committee (ref no.MU/AEC/VOLXIII/96) for Tanzania; and the Ethics Committee for Health Research in Burkina for Burkina Faso.

## Results

### Document review

Seven documents (Burkina Faso, n = 2; Ghana n = 2; Tanzania n = 3) were identified as CPGs for maternal care corresponding to the contents of WHO PCPNC sections B9-E in the three countries (Table 3). Guidelines in Burkina Faso and Ghana had been recently updated in 2009 and 2008 respectively, whereas Tanzanian guidelines were undergoing revisions but had not yet been published at the time of the review in 2010. The antenatal care (ANC) cards given to pregnant mothers provide a multi-purpose record form and although it is debatable whether they should be classified as CPGs, they are perceived to be the most available and used protocols by front line health workers in all three countries.

**Table 3:** Overview of reviewed national clinical practice guidelines for maternal care including an assessment of their format

	Name of Guideline	No. of pages	Format	
	
			Usability	Applicability
**Burkina** **Faso**	Protocoles De Santé De La Reproduction - Santé de la femme et du nouveau-né de moins de sept (7) jours (2009)	145	Low: text written in narrative format, difficult to navigate and find information	Low: no clear algorithms for decision making

	Carnet de Santé (Antenatal card) (No publication date)	35	Medium: record for ANC visits and delivery, checklists available but limited	Low: no algorithms for decision making, no partograph to monitor intra-partum care

**Ghana**	National Safe Motherhood Service Protocol (2008)	128	High: easy to navigate and find information	High: clear algorithms for decision-making and managing conditions and complications of pregnancy, delivery and postpartum period at different levels of care

	Maternal Health Record Book (Antenatal card) (No publication date)	15	Medium: checklists and record for antenatal, delivery and postpartum care	Medium: no algorithms for decision making, partograph to monitor intra-partum care included

**Tanzania**	RCH4 (Antenatal card) (2008)	5	Medium: checklists and record for antenatal, delivery and postpartum care	Medium: no algorithms for decision making, partograph to monitor intra-partum care included

	Emergency Obstetric Job Aid (2005)	41	Medium: easy to navigate but organised by obstetric condition rather than symptoms	High: clear algorithms for managing complications during pregnancy, delivery and postpartum period at different levels of care

	Focused Antenatal Care, Malaria and Syphilis in Pregnancy - Orientation Package for Service Providers (2002)	146	Low: text written in narrative format, no index, difficult to navigate and find information	Low: no clear algorithms for decision making

Overall, the contents of the national guidelines were similar to those of the WHO PCPNC. The few differences observed included: lack of a specific section on the management of ABC (Airway Breathing Circulation) in the Burkina Faso and Tanzanian guidelines; and deficiencies in guidance on how to respond to problems immediately postpartum in the Burkina Faso and Ghanaian guidelines (Table 4).

**Table 4:** Content comparison between the WHO PCPNC guidelines and the national clinical practice guidelines for maternal care (not including ANC cards)

Pregnancy, Childbirth, Postpartum and Newborn Care: A Guide to Essential Practice (WHO 2006) Guideline sections analysed:	Burkina Faso 'Protocoles de Santé de la Reproduction-Santé de la femme et du nouveau-né de moins de sept (7) jours'(November 2009)	Ghana 'National Safe Motherhood Service Protocol'(December 2008)	Tanzania 'Focused Antenatal care, malaria and syphilis in pregnancy (FANC)' (July 2002) 'Emergency Obstetric Job Aide (OJA)' (June 2005)
**Chapter B 9-17:** **Emergency Treatments for the woman**	Difference compared to WHO guidelines: No section on ABC (Airway, Breathing, Circulation). Malaria and infections in pregnancy not mentioned.	Difference compared to WHO guidelines: Nothing specific on malaria.	Difference compared to WHO guidelines: No section on ABC.

**Chapter B 19-21:** **Bleeding in early pregnancy and post-abortion care**	**Contains all points in WHO guidelines**	**Contains all points in WHO guidelines**	Difference compared to WHO guidelines: Bleeding only mentioned after week 28 of pregnancy

**Chapter C: Antenatal care**	Difference compared to WHO guidelines: Checking for anaemia and syphilis are only 'desired' examinations. No information on how to counsel women choosing home births without skilled attendants.	Difference compared to WHO guidelines: No emphasis on rapid assessment to exclude emergencies.	**Contains all points in WHO guidelines**

**Chapter Labour and Delivery**	**D: **Difference compared to WHO guidelines: No clear information on how to respond to problems immediately postpartum. Recommended return visits on 6^th ^to 8^th ^day of delivery (WHO 2^nd ^to 3^rd ^day).	Difference compared to WHO guidelines: Nothing about preterm delivery, severe anaemia on admission or how to respond to the following problems immediately postpartum: temperature > 38 degrees, pallor, stillborn baby.	Difference compared to WHO guidelines: OJA covers all WHO points, but does not describe labour in different stages

**E: Postpartum care**	**Contains all points in WHO guidelines**	Difference compared to WHO guidelines: HIV, breathing difficulties, postpartum depression not mentioned.	**Contains all points in WHO guidelines**

The review of guidelines' format revealed variations in the levels of usability and applicability between national CPGs in the three countries. Overall, the Ghanaian 'National Safe Motherhood Service Protocol' was the only CPG that had a high level of both usability and applicability, containing clear algorithms for clinical decision making at all levels of care and for all the stages of pregnancy, delivery, and the postpartum period. The Burkina Faso guidelines and the Tanzanian ANC guidelines had the lowest levels of both usability and applicability with mainly narrative text sections mixing background information and treatment recommendations and containing few algorithms for decision making. In these guidelines, navigation was cumbersome and guidance on problem solving difficult to find. On the other hand, the Tanzanian Emergency Obstetric Job Aid displayed a high level of applicability and medium level of usability, containing clear algorithms and flow charts for decision making, but arranged according to obstetric diagnosis rather than symptoms. All ANC cards had a medium level of usability with the Ghanaian and Tanzanian ones including detailed checklists for antenatal, delivery, and postpartum care. Checklists in the Burkina Faso ANC card were more limited and did not include postpartum care or a Partograph, which were included in the Ghanaian and Tanzanian ANC cards. None of the ANC cards included algorithms for decision making, lowering their level of applicability and use for clinical decision making.

### Key informant interviews

Analysis of the 28 interviews from Burkina Faso, Ghana and Tanzania identified three overarching themes: development of national CPGs for maternal health, health workers' access to guidelines, and health workers' use of guidelines. The first theme was part of the initial interview guide, whereas the second two emerged as a result of the semi-structured nature of interviews. Within each theme several categories emerged. Themes, categories, and their definitions are summarised in Table 5 and further described and illustrated with quotes below.

**Table 5:** Interview themes, categories and definitions

Themes	Categories	Definitions
**Development of national maternal health** **guidelines**	· Development Process	· the steps taken to develop and produce guidelines
	· Stakeholder participation	· level of involvement of stakeholders in guideline development
	· National guidelines' relation to WHO recommendations	· how contents of national CPGs compare with WHO PCPNC guidelines

**Health workers' access to guidelines**	Perceived access barriers	
	· Distribution	· physical distribution of printed guidelines
	· Staff mobility	· frequent changes of work-place displaces guidelines from health facilities
	· Health workers' participation in training courses	· course curricula frequently used as CPGs, health workers dependent on courses to obtain up-to-date guidelines
	Perceived solutions to improve access:	
	· Pocket sized guidelines	· personal portable guidelines for every health worker
	· Wall posters	· guidelines in a poster format increases availability for everybody working in the health facility

**Health workers' use of guidelines**	· Low levels of guideline adherence	· perception among key informants of an overall low use of guidelines by front-line health workers
	Perceived reasons for low guideline adherence:	
	· Attitudes towards continuing education	· health workers do not usually update their knowledge independently from organised training
	· Effects of training	· limited change in clinical practice following courses
	· Format of guidelines - lack of usability	· presence of flow-charts, algorithms etc.
	· Negative beliefs about using guidelines during patient consultations	· perception that patients' trust will be undermined if health workers use CPGs during consultations

### Development of national clinical practice guidelines for maternal health

In all three countries, the development of maternal health guidelines was reported to be carried out in cooperation between Ministries of Health and key stakeholders including UN organisations, NGOs, clinicians, professional associations, and to some extent health representatives from districts and regional levels as well as universities. A participatory approach seemed to be favoured by the key informants:

'... We do an initial draft; we call key stakeholders who make inputs about two or three times. In addition, the final version, we organize bigger stakeholders meeting to take inputs from key organizations and individuals.' (Medical doctor (MD), government level, Ghana)

In Tanzania and Burkina Faso, opinions however diverged as to whether sufficient participation had been achieved. In Tanzania, there was also disagreement on whether the new revision of antenatal care guidelines had been finalised and approved and whether they were in line with the WHO PCPNC guidelines:

'The process is sufficiently participatory.' (MD, government level, Burkina Faso)

'Heads of districts are involved although one may say that they are not adequately involved. It is for the validation of the document but not for the development.'(MD, district level, Burkina Faso)

'The revision was not participatory, that I have to tell you upfront...so basically those materials for the updated ANC need to be looked at to see if it is in line with this [WHO PCPNC].'(Program officer, international NGO, central level, Tanzania)

The WHO is a central partner for the development of guidelines in all three countries. No key informants believed that there were any major differences in content between WHO recommendations and national protocols. The recently updated Tanzanian Essential Newborn Care guidelines (not included in the document review) had been entirely based on WHO PCPNC. In Ghana, key informants stressed that the WHO guidelines constitute one but not the only source used as a basis for national protocols. In terms of the adaptation of the WHO generic guidelines, one key informant from Burkina Faso expressed:

'We are obliged to adapt to our context but not too far from what is advocated because we need quality care.'(MD, government level, Burkina Faso)

### Health workers' access to guidelines

In Burkina Faso, distribution of guidelines was perceived as a problem and the general view among key informants was that health workers' access to guidelines is limited:

'There is an effort made to have partners reproduce documents... But, often, they are kept stored in storehouses.' (MD, international NGO, central level, Burkina Faso)

'Between planning and having means, there is a gap. There is a big gap. We plan, develop [guidelines], and when it comes to implementation, we are not able to sufficiently mobilize resources.' (MD, government level, Burkina Faso)

In Tanzania, several versions of maternal health guidelines circulate. There are separate guidelines for antenatal care, postnatal care, PMTCT (Prevention of Mother to Child Transmission of HIV) and family planning. Mostly, health workers' access to materials depends on if they have recently attended a training course and on what subject:

'They [health workers] use everything what they get! Because even the job aide it is not everywhere. So those who have been trained in LSS [Life Saving Skills Curriculum in Emergency Obstetric Care], those [course materials] are their job aides.' (Program officer, international NGO, central level, Tanzania)

In Ghana, health workers' access to guidelines was not brought up during the interviews.

One reason related to poor access to guidelines in Burkina Faso was identified as staff mobility, both at district and at primary care level. Health workers consider guidelines as personal possessions and there is no inventory of what guidelines should be in place in each health facility:

'People transferred move with the documents instead of making copies and leaving the original document. There is no service transfer with an inventory set up.' (MD, government level, Burkina Faso)

As an effort to solve this problem in Burkina Faso, 7,000 copies of Emergency Obstetric and Newborn Care guidelines were to be printed in 2010 in a pocket size format. The same idea was also brought forward by a Tanzanian NGO. Another strategy perceived as a solution to the limited availability of guidelines was the use of wall posters:

'We were asked to make posters to be posted in different rooms... It helps the worker to take the right decision only after a glance.' (MD, government level, Burkina Faso)

### Health workers' use of guidelines

In all three countries, key informants expressed doubts as to whether guidelines are used and followed by health workers in practice. This was also confirmed by the few interviews with service providers:

'...my fear is, it [the guideline] may not be used at all.' (Reproductive health officer, international NGO, central level, Ghana)

'As for the conducting of the delivery and then after that the postnatal care, that one I know it as a midwife, but for the actual laid down guideline, I don't know.' (Health worker, district level health facility, Ghana)

In Burkina Faso, reasons identified for poor guideline adherence stem from health workers' initial vocational training as well as from their attitudes towards continuing education.

Having left pre-service training, new guidelines are not endorsed unless you attend a new course or orientation:

'But one must admit that the guidelines are not applied and this is due to the training of health workers themselves who come out of training schools with lacks.' (MD, district level, Burkina Faso)

'The issue of health workers requires a perpetual search of new knowledge or an updating. If at the end of the basic training at school, we do not open any document and we only expect workshops, there will be a problem.' (MD, government level, Burkina Faso)

The challenge of guideline implementation and the often disappointing outcomes of training courses were illustrated by a comment from a Tanzanian NGO worker:

'... there were people trained by the ministry before we moved there [district hospital] and yet when we came there, nobody had followed them [the guidelines], they had gone back to what they were doing before, they were not practising what they had been taught.' (Program manager, international NGO, central level, Tanzania)

In both Ghana and Tanzania, key informants expressed the lack of user-friendliness of guidelines as a probable reason for low adherence. The lack of flow charts, algorithms, and clear steps for how to manage different conditions is especially a problem with the training packages commonly used as guidelines by health workers in Tanzania:

'But unfortunately they [training curriculums] have not been followed up by flow charts, these are not there, so these need to be extracted and developed. For example, if you manage eclampsia, what are the steps?' (Program officer, international NGO, central level, Tanzania)

'Maybe the guidelines were not easy to follow.' (Reproductive health officer, international NGO, central level, Ghana)

In Burkina Faso, another reason for the perceived limited use of guidelines was negative beliefs about using guidelines during patient consultations:

'... We have a problem on how to have permanently documents laid on consultation table. First of all, people think that it devaluates the fact of consulting with a paper before the patient while one must ensure what they are going to do is right.' (MD, government level, Burkina Faso)

## Discussion

While all three countries in this study incorporate WHO recommendations when producing national CPGs for maternal health, there are variations in the levels of guidelines' usability and applicability (Figure 1). In all three countries, the use of guidelines by health workers in daily practice is perceived to be limited. The cross-country comparison identifies barriers to the potential impact of CPGs on improving quality of care for mothers in Sub Saharan Africa. It emphasises the need to prioritise the format of guidelines to increase their usability and applicability and to consider implementation strategies as integral to their development processes.

No key informants believed that there were any major differences in content between the national guidelines for maternal health and the WHO recommendations. This impression was largely supported by the document review. Indeed, the WHO acted as a main partner in maternal health CPG development in all three countries. This reflects the recommendation to the WHO to support local adaptation processes presented in a study from 2008 where factors associated with successful promotion of the use of research evidence in health policy development were investigated [41]. The same study however identified the often lengthy and cost-intense nature of methods used to produce CPGs as a main weakness limiting both quantity and quality of CPGs as well as the frequency of their updates [41]. It is therefore possible that the resources spent on CPG development and adaptation of contents is given too high priority. Overall, the quality of the content and evidence base for maternal health CPGs in the three study countries seem to be the least important barrier to their impact on quality improvement in maternal health [41].

The document review revealed variations in the number of documents covering the contents of the WHO PCPNC guidelines between the three countries. In Burkina Faso and Ghana, there was one main guideline, whereas in Tanzania the same content was covered by two different documents. In addition, the ANC cards given to pregnant mothers in all three countries provide the most accessible and used guideline by front line health workers. In Tanzania, several other guidelines for different aspects of maternal care were also identified during the interviews. This division of documents could have contradicting effects. Clearly specified user groups with separate guidelines for different levels of care could increase the use of guidelines through enhancing features of applicability and adaptability (Figure 1) [21]. As distribution of guidelines and their use by health workers in practice is a recognised challenge [11,14,17], this division of documents could however limit use further through added complexity and duplication of implementation processes. One possible explanation for the multitude of maternal health guidelines observed in Tanzania could be a lack of coordination of multiple donors supporting parallel interventions, a common phenomenon in the field of maternal health [42].

The cross-country comparison of guidelines displayed marked differences in their levels of usability and applicability (Figure 1) [21]. CPGs from Burkina Faso as well as the Tanzanian focused ANC guidelines were difficult to navigate, and the recommendations were often non-specific, reducing their applicability. The Ghanaian guidelines included clear algorithms to aid decision making, as did the Tanzanian Emergency Obstetric Job Aid. Research has shown that guideline characteristics, such as clarity of advice and easy-to-follow format, influence their use by health workers in practice [16,21,38,43]. It is therefore plausible that even where CPGs are available to health workers in Burkina Faso and for antenatal care in Tanzania, their use and hence impact will be limited (Figure 1) [21]. In high-income countries, the use of ICT to enhance guideline implementation has shown promising results. CDSSs can provide a high level of both usability and applicability, as well as adaptability through links to individual patient records and different versions for different users [20,23-25]. The use of such systems in low-income settings could be of potential value but issues related to electricity-supply, computer literacy of staff, opportunity costs, and sustainability in these environments need to be carefully considered [22,44]. The implementation of a computerised CDSS in the study countries, as part of the QUALMAT project, will therefore be an important trial.

In our study, the distribution of CPGs and health workers' access to them was perceived to be a problem, especially in Burkina Faso and Tanzania. This is an experience shared by many other low-income settings, largely due to the inadequate resource allocation for guideline implementation [17,45]. The need to 'be attentive to implementation considerations, even if implementation is not a remit' was indeed identified as one of seven recommendations to organisations involved in supporting the use of research evidence in developing health policy including CPGs in low and middle income countries in a study from 2008 [41]. The low frequency of guideline use by front-line health workers was expressed as a problem in all countries. This is of particular concern as the main purpose of CPGs is to improve quality of care that is often poor in low-income settings [10,16]. At the same time, it implies an untapped potential to increase the skills of health workers and reinforces the importance of guideline characteristics, such as usability and applicability, with a potential to improve their implementation and raise the quality of care (Figure 1) [21].

### Methodological considerations

The generalisability of the insights gained in this paper is increased by the use of cross-country comparisons [27]. Results were triangulated through the use of document reviews, key informant interviews, and informal discussions with maternal health experts. Because different research teams carried out the interviews in the three countries and the interview guide used was semi-structured, there were some variations in the issues discussed and therefore differences in the data obtained for analysis. We still believe that the use of data collection tools was similar enough in the three countries to make a cross-country comparison relevant and useful. There was no pre-specified number of interviews per country. The numbers carried out rather reflected the initial study objectives of identifying all national maternal health guidelines, their development processes and content compared to WHO recommendations, which was achieved through purposive sampling. It is therefore unlikely that the findings have been affected by the fact that there were fewer interviews in Tanzania. Also, due to the initial study objectives, no peripheral key informants or health workers were interviewed in Tanzania and overall only seven front-line health workers were interviewed in Burkina Faso and Ghana. This has to be considered when interpreting the perceptions of use of guidelines in the three countries, as a larger number of front-line health workers would have been included had this been an original main objective of the study.

### Conclusions and policy implications

Our cross-country study suggests that the major barrier for CPGs to positively impact on quality improvement in maternal care in Sub Saharan Africa is not their poor quality of content or lack of evidence base. Rather, the format of guidelines and the deficiencies in their implementation, negatively affecting availability and use by health workers, constitute major bottlenecks. While national adaptation processes of generic WHO guidelines are important for approval and attainment of local ownership, which may be important for CPG uptake and use, our results suggest that focusing on content only is not enough. It is clear that if followed and used in practice, the identified guidelines would improve quality of care, with a positive impact on maternal morbidity and mortality. Improving implementation strategies, including dedicated attention to guidelines' format is therefore imperative, and could provide another way to attain local ownership. Although further research is needed to assert the importance of these aspects, we suggest that they should be considered when developing and adapting new guidelines.

## Abbreviations

ANC: Antenatal care; CDSS: Clinical Decision Support System; CPG: Clinical Practice Guideline; ICT: Information and Communication Technology; MD: Medical doctor; MDG 5: Millennium Development Goal 5; MMR: Maternal Mortality Ratio; NGO: Non-governmental organisation; PCPNC: 'Pregnancy Childbirth Postpartum and Newborn Care: A Guide for Essential Practice'; SSA: Sub Saharan Africa; WP2: Work package 2 (of the QUALMAT project); QUALMAT: Quality of Maternal and Prenatal Care.

## Competing interests

The authors declare that they have no competing interests.

## Authors' contributions

UB participated in the design of the study, carried out data collection in Tanzania, transcribed the Tanzanian interviews, analysed and interpreted the data, drafted and revised the manuscript. GT conceived and participated in the design of the study, data analysis, interpretation of results and writing of the manuscript. MS conducted and transcribed the interviews in Burkina Faso. BK participated in the design of the study and the coordination of the fieldwork in Burkina Faso. AB participated in the interpretation of results and writing of the manuscript. JW participated in the design of the study, the coordination of the data collection in Ghana and the interpretation of results. RM participated in the design of the study, participated and coordinated the data collection in Tanzania, and participated in the data interpretation. SM participated in the design of the study, document review of guidelines and the interpretation of results. LLG participated in the design of the study and the interpretation of results. JE participated in the design of the study, coordinated the data collection, participated in data collection in Burkina Faso and Ghana, and participated in data analysis, interpretation of results and writing of the manuscript. All authors read and approved the final manuscript.

## Supplementary Material

Additional file 1 Guide for semi-structured interviews with key informants. (Contains the questions used for topics (1) and (3) as explained in the methods section.). Click here for file

Additional file 2 Data extraction check list for content comparison between national CPGs for maternal health and WHO PCPNC. (Contains all the sub-elements of sections B9-E, which were used for the content comparison.). Click here for file
