# Supplementary material for: 'How to know what you need to do': a cross-country comparison of maternal health guidelines in Burkina Faso, Ghana and Tanzania
Source: Implement Sci. 2012 Apr 13;7:31. doi: 10.1186/1748-5908-7-31 (PMC3372446; doi:10.1186/1748-5908-7-31)
Supplement: Additional file 1 — Guide for semi-structured interviews with key informants. (Contains the questions used for topics (1) and (3) as explained in the methods section.). [file 1748-5908-7-31-S1.DOCX]

# Additional file 1: Guide for semi-structured interviews with key informants

**Introduction**

Introduce QUALMAT and go through consent procedure.

**Questions**

**A: 1 National CPGs for maternal health**

- Are you aware of the National Reproductive Health Service Protocols?
- Do you know of the procedures for writing and formulating these protocols? Were you involved in the process?
- What were the procedures for writing and formulating these protocols? (e.g. consultations, meetings etc.)
- What were they based on? (Probe for other guidelines, e.g. WHO guidelines, national previous guidelines etc., latest research)
- If based on the WHO guide “Pregnancy, Childbirth, Postpartum and Newborn Care – A guide for Essential practice 2006” (WHO PCPNC):

- What differences are there to the WHO PCPNC?

- Why were those differences decided upon?

- If not based on WHO PCPNC:
- Are you aware of the WHO PCPNC?
- Why was the WHO PCPNC not used?
- Are there any plans to use WHO PCPNC?

**A: 3 Current maternal health projects and initiatives**

- Which activities / initiatives are you aware of in the field of maternal healthcare?

(Prompts: Revision of current policies? Any on-going plans? Projects through NGOs or other organisations? research? )

- Are you aware of any initiatives/research within the xxxx *[study]* and xxxxx *[control]* districts?
- Are you aware of any initiatives/research into electronic tools (for example in HIV/AIDS)?
