# Supplementary material for: 'How to know what you need to do': a cross-country comparison of maternal health guidelines in Burkina Faso, Ghana and Tanzania
Source: Implement Sci. 2012 Apr 13;7:31. doi: 10.1186/1748-5908-7-31 (PMC3372446; doi:10.1186/1748-5908-7-31)
Supplement: Additional file 2 — Data extraction check list for content comparison between national CPGs for maternal health and WHO PCPNC. (Contains all the sub-elements of sections B9-E, which were used for the content comparison.). [file 1748-5908-7-31-S2.DOCX]

**Additional file 2: Data extraction check list for content comparison between national CPGs for maternal health and WHO PCPNC**

| **WHO PCPNC**  **chapters** | **Data extraction elements** |
| --- | --- |
| **B 9-17: Emergency Treatments for the woman** | Airway, breathing, circulation  Bleeding  Considerations for eclampsia and pre-eclampsia  Infection  Malaria  Referral |
| **B 19-21: Bleeding in early pregnancy and post-abortion care** | Examination  Preventive measures  Advice |
| **C: Antenatal care** | Assess the pregnant woman: pregnancy status, birth and emergency plan  Check for pre-eclampsia  Check for anaemia  Check for syphilis  Check for HIV status  Respond to observed signs or volunteered problems  Give preventive measures  Advise and counsel on nutrition and self-care  Develop a birth and emergency plan  Advise and counsel on family planning  Advise on routine and follow-up visits  Home delivery without a skilled attendant  Assess eligibility of ARV for HIV-positive pregnant woman |
| **D: Labour and Delivery** | Examine the woman in labour or with ruptured membranes  Decide stage of labour  Respond to obstetrical problems on admission  Give supportive care throughout labour  First stage of labour  Second stage of labour: deliver the baby and give immediate newborn care  Third stage of labour: deliver the placenta  Respond to problems during labour and delivery  Care of the mother and newborn within first hour of delivery of placenta  Care of the mother one hour after delivery of placenta  Assess the mother after delivery  Respond to problems immediately postpartum  Give preventive measures  Advise on postpartum care  Counsel on birth spacing and family planning  Advise on when to return  Home delivery by skilled attendant |
| **E: Postpartum care** | Postpartum examination of the mother (up to 6 weeks)  Respond to observed signs or volunteered problems |
